# Valorization of Biomasses from Energy Crops for the Discovery of Novel Thermophilic Glycoside Hydrolases through Metagenomic Analysis

**DOI:** 10.3390/ijms231810505

**Published:** 2022-09-10

**Authors:** Roberta Iacono, Andrea Strazzulli, Rosa Giglio, Federica Bitetti, Beatrice Cobucci-Ponzano, Marco Moracci

**Affiliations:** 1 Department of Biology, University of Naples “Federico II”, Complesso Universitario di Monte S. Angelo, Via Cupa Nuova Cinthia 21, 80126 Naples, Italy; 2 Task Force on Microbiome Studies, University of Naples “Federico II”, 80138 Naples, Italy; 3 Institute of Biosciences and BioResources—National Research Council of Italy, Via P. Castellino 111, 80131 Naples, Italy

**Keywords:** CAZymes, extremozymes, archaea, enrichments

## Abstract

The increasing interest for environmentally friendly technologies is driving the transition from fossil-based economy to bioeconomy. A key enabler for circular bioeconomy is to valorize renewable biomasses as feedstock to extract high value-added chemicals. Within this transition the discovery and the use of robust biocatalysts to replace toxic chemical catalysts play a significant role as technology drivers. To meet both the demands, we performed microbial enrichments on two energy crops, used as low-cost feed for extremophilic consortia. A culture-dependent approach coupled to metagenomic analysis led to the discovery of more than 300 glycoside hydrolases and to characterize a new α-glucosidase from an unknown hyperthermophilic archaeon. Aglu1 demonstrated to be the most active archaeal GH31 on 4Np-α-Glc and it showed unexpected specificity vs. kojibiose, revealing to be a promising candidate for biotechnological applications such as the liquefaction/saccharification of starch.

## 1. Introduction

Extremozymes from thermophilic microorganisms possess notable properties, such as thermostability, and robustness in catalytic activity, making them interesting for a plethora of commercial applications such as the chemical industry, bioremediation and biorefinery [1,2,3,4].

Global annual lignocellulosic generates nearly 731 and 709.2 million tons of wastes of rice straw [5] and wheat straw [6], respectively; and 5 and 200 billion tons of crop residue waste [7] and plant biomass [5], respectively. Lignocellulosic biomasses are rich in sugars, lipids, proteins, and vitamins and have a chemical composition that can support microbiological growth [8]. Indeed, lignocellulosic biomasses from agricultural crops or wood have been utilized as substrates in fermentation processes (e.g., as part of culture media) to yield commercially relevant compounds such as ethanol [9,10], organic acids [11,12], enzymes [13,14], polymers [15], hydrogen [16], especially as alternatives of starch producing crops, thereby avoiding competition with food production [17,18].

These feedstocks can have different origins, depending on the biomass availability in different geographical regions in order to cut the costs and CO_2_ emissions due to their transportation in the biorefineries plants. In a study performed in Italy, it has been demonstrated that the plant and the transportation of biomass are the main contributors to the production of CO_2_, accounting for about the 38% and 34% of the total, respectively [19].

*Arundo donax* and *Cynara cardunculus*, two native Mediterranean plant species, have been recently identified as potential energy crops in this area. *C. cardunculus*, also known as wild cardoon or Castilian thistle, is a perennial herbaceous species with an annual growth cycle. The branched stalks of cardoon account for about 40% of total dry biomass and are composed generally of 20–30% of hemicelluloses (made up of arabinoglucuronoxylan), 35–45% of cellulose and 10–20% of lignin [20]. In addition, in *C. cardunculus* inulin is often stored in specialized organs, such as taproot, bulbs, and capitula and used as a carbon source during regrowth and sprouting in the spring [21]. *A. donax*, also known as giant reeds, is a perennial rhizomatous wetland grass with high content of cellulose, hemicellulose (composed of arabinoglucuronoxylan), and lignin, by 31%, 35% and 18%, respectively [22]. In addition, the most abundant non-structural carbohydrates in this energy crop are sucrose and starch [23].

Due to the different types of polysaccharides present in these herbaceous species and to their structural complexities, complete enzymatic deconstruction requires the synergistic action of several carbohydrate active enzymes (CAZymes). “Omics” approaches have provided a powerful tool for the discovery of new extremozymes from nature [24]. In fact, most extremophilic microorganisms are recalcitrant to lab cultivation and isolation approaches [4], therefore, culture-independent metagenomic strategies are promising approaches to assess the phylogenetic composition and functional potential of microbial communities living in extreme environments [25]. Recently, our group reported on the metagenomic analysis of the microbial communities populating the Pisciarelli hot springs (Naples, Italy, 40°49′45.1″ N; 14°8′49.4″ E) [26,27], identifying the repertoire of carbohydrate active enzymes (CAZome) produced by the microbial community of extremophiles populating this environment [27]. Pisciarelli’s microbiome showed a huge number of genes encoding putative CAZymes, which include glycoside hydrolases (GHs), carbohydrate esterases (CEs), polysaccharide lyases (PLs), and auxiliary activities (AAs) [27], which are classified in the CAZy database (www.cazy.org; accessed on 9 August 2022) [28].

The main aim of this study was to select new CAZymes by enriching Pisciarelli’s samples on *A. donax* and *C. cardunculus* as low-cost rich carbon sources as an alternative to the common culture media, and as possible inducers of growth of specialized microorganisms. Since Pisciarelli solfatara is mainly populated by Archaea strains that are often difficult to isolate in laboratory conditions, we follow a metagenomic approach to analyze the efficacy of the enrichments and to identify the enzymes of interest. We report here that this approach led to the discovery of hundreds of new Glycoside Hydrolases (GHs) and the characterization of a thermophilic and thermostable α-glucosidase from an unknown hyperthermophilic archaeon.

## 2. Results

### 2.1. Enrichments

The mud/water sample collected in Pisciarelli solfatara (Pool1, pH 5.5 and 85 °C) in March 2012 [27] was incubated in Basal Salt Medium (BSM) at pH 1.8 [29], Brock salt medium pH 3.5 [30], or Pyrobaculum (PYR) salt medium pH 7.0 [31] supplemented by yeast extract, tryptone and sucrose (YTS) 0.1% as an initial carbon source. *A. donax* (giant reed) pretreated by steam-explosion (0.15% *w*/*v*) was used in the first trial as alternative rich carbon source. Only the microbial enrichment in Brock medium with pretreated *A. donax* or with YTS 0.1%, showed an increase in optical density, while no microbial growth was observed in BSM and PYR salt media containing the same sources of energy (Figure S1).

After this preliminary trial, a fresh culture was set up by starting from the same environmental sample in Brock salt medium supplemented by YTS 0.1% as an initial culture broth. After 24 h, the culture was split into four sub-cultivations and YTS was substituted with *A. donax* or *C. cardunculus* (Thistle), both as pretreated and woodchip. Contextually, a further culture was performed by using YTS 0.1% as a carbon source. Growth was observed in all enriched cultures, except on pretreated Thistle. When the enrichments reached 0.4 OD_600_, total DNA was purified from the cultures as described in Materials and Methods, and fully sequenced.

### 2.2. Sequence Analysis and Microbial Composition

**Figure 1:**
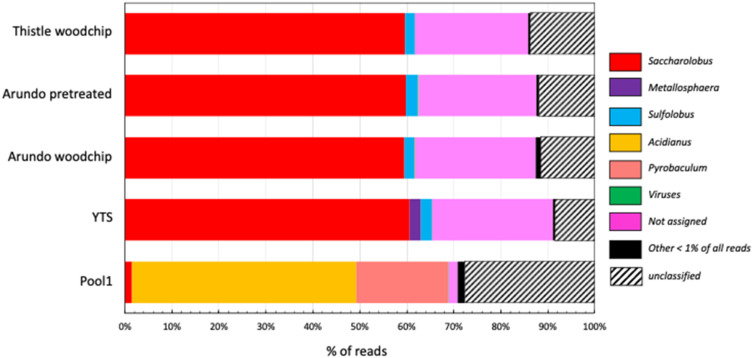
Taxonomic annotation at the genus level.

The microbial composition of all the enriched samples (Arundo woodchip, Arundo pretreated, Thistle woodchip, and YTS), was analyzed by performing a metagenomic sequence-based approach by Illumina (San Diego, CA, USA) HiSeq sequencing (Table S1), followed by community diversity analysis based on assigning reads to known microorganisms (Figure 1).

Interestingly, in all samples, among the known genus, the most abundant is *Saccharolobus* (~60%) followed by *Sulfolobus* ranging from 2.5% in the sample containing Arundo pretreated to 2.0% in Thistle. More specifically, the reads assigned to the genus *Saccharolobus* are related to the species *S. solfataricus* (57%), *S. shibatae* (0.6%), and *S. caldissimus* (0.1%). Differently, the genus *Metallosphaera* is present only in the YTS sample (2.4%) and, possibly it has been negatively selected by the other biomasses.

The carbon sources in the enriched samples drove a dramatic selection of the microbial community present in the original environmental sample of Pool1 (Figure 1). In fact, while Pool1 was dominated by the genera *Acidianus* (48%) and *Pyrobaculum* (19%), in the enriched samples the first one is present at 0.2% while the latter is completely absent.

It is worth noting that in all the enriched samples at the genus level, the percentage of not assigned reads and sequences that can be assigned to ranks higher than genera is ~25%, while at the ranks of phylum, class, order, and family, the percentage of unassigned reads is <0.1%. This indicates the presence of microorganisms belonging to the *Sulfolobaceae* family but different from the genera present in the Refseq NCBI database (Figure S2). In addition, in all the enriched samples, there are reads that could not be taxonomically classified (termed unclassified in Figure 1) representing 8.5%, 11.5%, 12%, and 13.8% of the reads of YTS, Arundo woodchip, Arundo pretreated, and Thistle woodchip, respectively, while in Pool1 these represented 28%.

The reads of each sample were assembled individually and 1338 contigs (≥500 bp) were obtained from YTS, 1751 from Arundo woodchip, 1368 from Arundo pretreated, and 549 from Thistle woodchip. Subsequently, the analysis of Open Reading Frames (ORFs) allowed the identification of 9947 in YTS, 8334 in Arundo woodchip, 6439 in Arundo pretreated, 4300 in Thistle woodchip, and 14,934 in Pool1 encoding sequences for hypothetical proteins (Table S1).

### 2.3. CAZome Analysis, Cloning, Expression, and Purification of Aglu1

**Figure 2:**
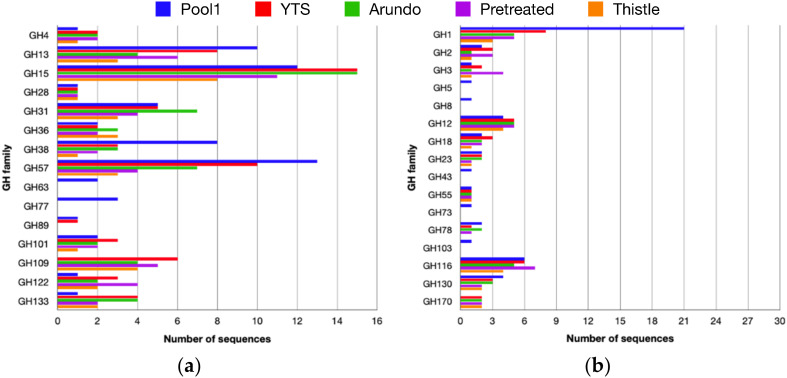
Distribution of the glycosidases active on axial (**a**) and equatorial (**b**) C1-OR bonds.

In order to evaluate if the enrichment on the different media modified the number and/or type of CAZymes, the previously identified ORFs were analyzed using the dbCAN2 pipeline [32]. The analysis revealed that among CAZymes, Glycosyl Transferases (GTs) (Figure S3a) represent the most abundant class (52% of the total number of CAZymes) grouped in 12 families, followed by GHs (39%) (Figure 2). In contrast, Carbohydrate Esterases (CEs) and Auxiliary Activities (AAs) (Figure S3b) are much less abundant (5% and 4%, respectively).

Among GHs, 23 families were identified in all samples, 14 of which were active on substrates with axial C1-OR bond (Figure 2a) and 11 on substrates showing equatorial C1-OR bond (Figure 2b) for a total of >300 sequences encoding for putative GHs. In general, selection produced a clear change in CAZymes composition in the four enrichments if compared to Pool1, as both number and type of families, and numerosity of enzymes. In particular, among the enzymes active on substrates with equatorial O-glycosidic bonds (C1-OR), different sequences belonging to families of exo- and endo-glycosidases active on hemicellulosic oligosaccharides such as xylan, xyloglucan, and mannan (GH1, GH3, GH12, and GH116) have been identified. Among the families grouping enzymes active on axial C1-OR bonds, 6 group exo- and endo-glycosidases were active on starch and maltodextrines (GH13, GH15, GH31, GH57, GH122, and GH133). It is worth noting that most of the GHs families active on substrates containing equatorial C1-OR bonds that have been found in the microbiomes growing on the four carbon sources tested were present in the original sample from Pool1. The number of sequences present in the GHs families active on equatorial C1-OR bonds from the enrichments was about the same as those found in Pool1. However, a clear selection was observed. Sequences from GH5, GH8, GH73, and GH103, which were present in Pool1, were absent in the enrichments and the number of sequences assigned as GH1 originally present in Pool1 was dramatically reduced upon enrichment. On the other hand, the sequences annotated in GH170 that could be identified in the microbiomes from all four enrichments were lacking in Pool1 and family GH3 showed 4 CAZyme sequences in Arundo pretreated vs. 1 in Pool1.

Among the selected GHs families active on axial C1-OR bonds, a higher variety was observed. Sequences in families GH122 and GH133 significantly increased in number if compared to those present in Pool1. GH109, which is absent in Pool1, showed several sequences in all four enrichments. In addition, sequences from two families, GH15 and GH31, grouping enzymatic activities involved in starch degradation, were more abundant in Arundo woodchip than in Pool1.

These observations suggested that enrichments produce a more significant selection on the CAZymes involved in starch degradation, rather than in lignocellulose hydrolysis. This prompted us to analyze in more depth the sequences encoding for starch-degrading enzymes. In particular, a specific gene of 2082 bp present in all samples, named *aglu1*, and encoding for a hypothetical protein (Aglu1) of 693 aa belonging to family GH31, was identified in a contig of 21,649 bp (Figure S4b).

The multialignment of Aglu1 against other characterized archaeal representatives of the GH31 family, including MalA and XylS from *S. solfataricus* [33,34], ST2525 from *S. tokodaii* [35], MalA from *S. acidocaldarius* [36], AglA from *P. torridus* [37], and Agla from *T. acidophilum* [38] confirmed the presence of several conserved regions. In particular, the highly conserved catalytic residues of GH31 family members strongly suggested that *aglu1* could encode for a functional enzyme (Figure S5).

**Figure 3:**
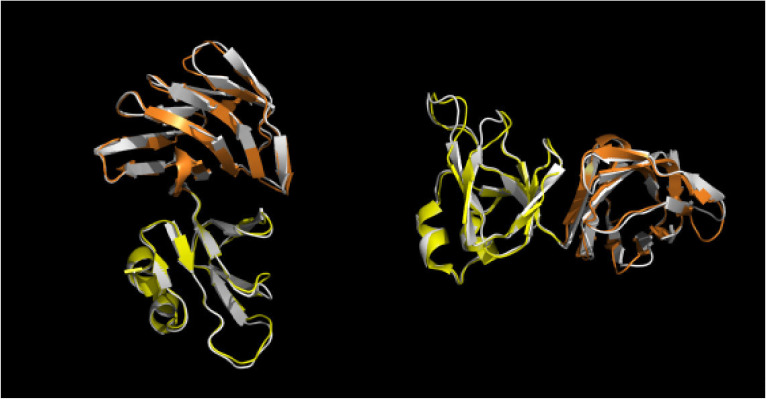
Close-up superimposition of *S. solfataricus* MalA C (yellow) and D (orange) domains and Aglu1 model (white).

To date, the crystallographic study of the *S. solfataricus* α-glucosidase MalA is notable as the first and sole experimental report of the 3-D structure of an archaeal (hyper)thermophilic GH31 [39], sharing 82% overall amino acid identity with Aglu1. In order to investigate the possible three-dimensional structure of Aglu1, a model was prepared using ColabFold [40] and compared to the MalA structure (PDB ID: 2G3M). As reported by Ernst and collaborators, MalA presents 4 domains (N, A, C, and D) and two subdomains (B and B’) [39] (Figure S6a). The Aglu1 3D model conserved all MalA domains and subdomains with an overall RMSD of 0.461Å (Figure S6b,c). However, detailed analysis showed specific differences in the sequence identity of the five domains. In particular, the N-terminal (N), the catalytic domain (A), and the subdomains B and B’, showed identities with MalA and other α-gluco and xylosidases of the GH31 family in the range of 86–90% [39]. Instead, the C and D domains of MalA and Aglu1 had significantly lower sequence identity (68% and 50%, respectively) (Figure 3). In particular, it has been suggested that the D domain, highly heterogeneous in the family, could be involved in the binding of starch substrate [41].

In order to understand if the observed structural diversity had functional implications, the gene *aglu1* was cloned in the pET20b expression vector and recombinantly expressed in *E. coli* BL21 (DE3) RIL. The recombinant Aglu1 was subsequently purified through six steps of purification, as described in materials and methods, with a final yield of 34% (1.25 mg/L of culture) and a purity of 95% (Table S2).

### 2.4. Molecular Mass Determination, pH and Temperature Dependence

SDS-PAGE analysis of the recombinant purified Aglu1 revealed the presence of a band at the predicted molecular weight of 80 kDa (Figure S7). In native conditions, Aglu1 showed a molecular mass of 462 ± 1.2 kDa, as demonstrated by size exclusion chromatography indicating a hexameric structure (Figure S8) as observed for MalA [39].

**Figure 4:**
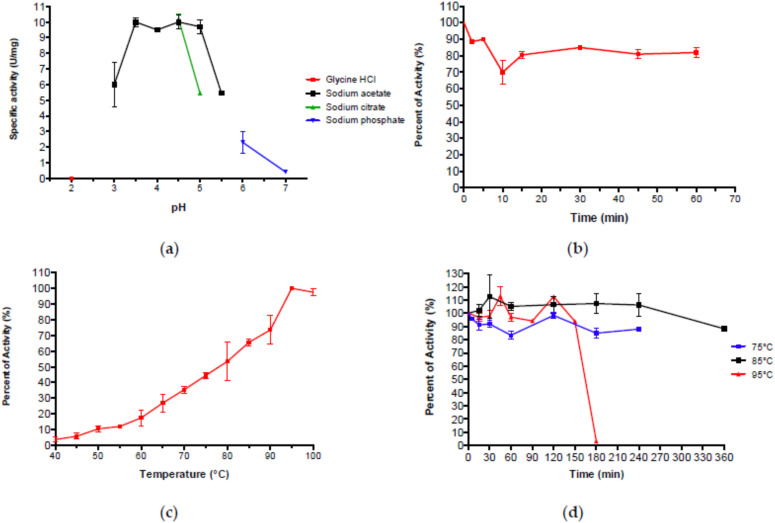
(**a**) Dependence of Aglu1 activity on pH. (**b**) Stability of the Aglu1 activity at pH 4.5. (**c**) Dependence of Aglu1 activity on temperature. (**d**) Heat stability of Aglu1.

Aglu1 standard assays were performed on pNp-α-Glc. The enzyme was optimally active in a sodium acetate buffer in the range of pH 3.5–5.0 (Figure 4a), as other thermophilic GHs showing a preference for slightly acidic pH [1,27,34,35,42]. PRED-TAT analysis indicated the absence of a signal peptide excluding that Aglu1 is a secreted enzyme. At pH 4.5, the enzyme retained 80% of its maximal activity up to 1 h at 65 °C (Figure 4b).

As expected for an enzyme isolated from an enrichment performed at 80 °C, Aglu1 showed its maximal activity (~20 U/mg) at 95 °C which was reduced to 26%, and 66% at 65 °C and 85 °C, respectively (Figure 4c), as observed in archaeal α-glucosidases from GH31 [33,34,36,37,38]. In addition, Aglu1 exhibited remarkable stability at 75 °C, 85 °C, and 95 °C maintaining 100% of activity after 240, 360 and 120 min, respectively (Figure 4d). These results demonstrated that Aglu1 is a highly thermostable acid α-glucosidase.

### 2.5. Substrate Specificity and Kinetics Parameters

Preliminary substrate screening on aryl-glycosides, malto-oligodextrins, trehalose, nigerose and polysaccharides (starch and glycogen) at 65 °C for 1 h revealed that Aglu1 was able to hydrolyze α-1,4-, α-1,3- and α-1,2 -glycosidic bonds with a decreasing specific activity on higher maltodextrin DP, starch, and glycogen. The enzyme was not active on trehalose (α-1,1-) and on the aryl-glycosides 4Np- α-Xyl, -α-Fuc, -α-Gal, -α-Man, -α-L-Ara. Standard activity at 85 °C was measured on the best substrates 4Np-α-Glc, maltose, maltotriose and kojibiose showing 22.2 ± 0.6 U/mg, 73.9 ± 1.3 U/mg, 73.3 ± 5.0 U/mg and 54.0 ± 3.0 U/mg, respectively.

To investigate the substrate specificity, kinetic parameters were determined on the best substrates at 85 °C. Aglu1 showed the highest specificity constant on maltotriose followed by kojibiose, maltose and 4Np-α-Glu (Table 1 and Figure S9).

**Figure 5:**
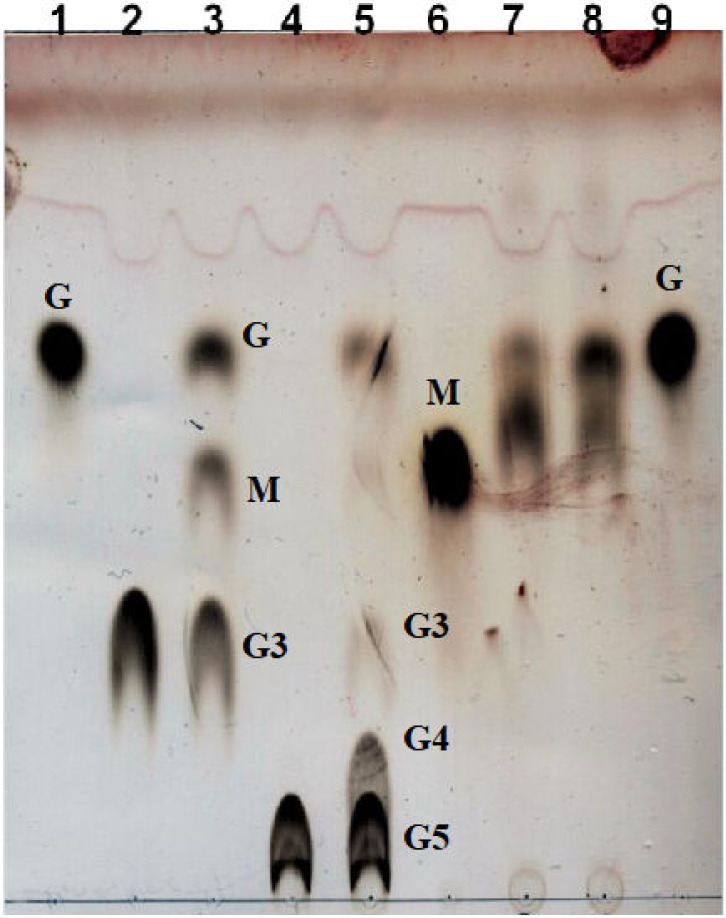
TLC analysis of the enzymatic assays on maltotriose, maltopentaose and nigerose. Lanes 1 and 9: Glucose. Lane 2: blank on maltotriose. Lane 3: Aglu1 on maltotriose. Lane 4: blank on maltopentaose. Lane 5: Aglu1 on maltopentaose. Lane 6: Maltose. Lane 7: blank on nigerose. Lane 8: Aglu1 on nigerose. G: glucose; M: maltose; G3: maltotriose; G4: maltotetraose; G5: maltopentaose.

The ability of Aglu1 to hydrolyze maltotriose (G3), maltopentaose (G5) and nigerose was further investigated by thin-layer chromatography (TLC). As shown in Figure 5 (lanes 3 and 5), the enzyme produced glucose and maltooligodextrins shortened by one unit compared to the substrate, revealing that the enzyme is exo-acting. In addition, Aglu1 showed weak activity on nigerose (Glc-α1,3-Glc), detecting a partial hydrolysis of the substrate after 1 h (Figure 5, lane 8).

## 3. Discussion

The study aims to take advantage of the metabolic potentiality of the microbial communities inhabiting the site Pool1 in Pisciarelli solfatara hot springs by enriching the microbial communities on biomasses of biotechnological interest and analyzing the CAZymes that could be selected. A recent metagenomic study on the microbial population of Pool1 showed a remarkably high set of CAZymes that captured our attention for their industrial applications [27]. Keeping this in mind, we set up lab enrichments on different carbon sources: commercial medium YTS and natural media from energy crops such as pretreated and woodchips of *A. donax*, woodchips of *C. cardunculus*. Our strategy was based on a metagenomic analysis of the enriched growths to avoid possible limitations due to the difficult isolation of archaeal strains.

It was expected that enrichments on recalcitrant biomasses could lead to the loss of major portions of the microbial population present in environmental samples [43]. Thus, to maintain the highest microbial diversity in the samples during the enrichment, a mild selective pressure for a short period was applied. More specifically, we used the growth media on which the genera, identified in the previous study [27] demonstrated the ability to grow [44,45,46,47]. Next, during the cultivation, the carbon source was slowly replaced by adding natural biomasses.

After lab enrichments, a remarkable reduction of the microbial diversity and a switch in the composition of the microbial population were observed. A dramatic decrease in the number of identified ORFs, passing from 14,934 in Pool1 [27] to a range between 9947–4300 in the enriched samples (Table S1), confirmed the strong selection of the microorganisms present in Pool1 after the enrichments. Moreover, Pool1 was dominated by *Acidianus hospitalis*, representing more than 50% of the entire microbiome [27] (Figure 1) while the enriched population was dominated by the genus *Saccharolobus* (mainly *S. solfataricus*), followed by *Sulfolobus* (which was only ~1.5% in Pool1) and by not assigned microorganisms (Figure 1 and Figure S2). This might reflect the ability of *S. solfataricus* to grow aerobically that might have outcompeted *A. hospitalis* [48]. Another strong selection might have occurred because of the carbon source used for the enrichment and the growing medium. *S. solfataricus* can grow on a variety of sugars, such as polysaccharides (cellulose, starch, dextrin), disaccharides (maltose, sucrose, lactose), hexoses (e.g., D-galactose, D-glucose, D-mannose and L-fucose), and pentoses (e.g., D-xylose and L-arabinose) [49], reflecting the abundance of CAZymes in *S. solfataricus* P2 (28 GHs belonging to 16 different families). In contrast, *A. hospitalis* shows only 19 GHs belonging to 10 different families [28] and, although being able to grow on yeast extract [50], the use of Brock medium and the aerobic conditions might have limited its growth on YTS medium.

The functional annotation of the ORFs identified in the analyzed samples revealed 267 CAZymes in the YTS medium sample, 208 in Arundo woodchip, 194 in Arundo pretreated, and 135 in Thistle woodchip representing ~3% of the total ORFs. Among the CAZymes, 312 sequences have been annotated as putative GHs belonging to 23 different families (Figure 2), 83 from the Arundo woodchip sample, 78 from the Arundo pretreated and 52 from the Thistle woodchip.

It is worth noting that the growth on media containing YTS and Arundo and Thistle biomasses resulted in a stronger selection toward CAZymes active on substrates containing axial rather than equatorial C1-OR bonds. In fact, the number of sequences for CAZymes active on the latter substrates in microbiomes obtained from the enrichments was similar and in one case much reduced if compared to the CAZymes’ sequences composition of Pool1 (Figure 2). Remarkably, the number of GH1 sequences is halved after the enrichments. This can be explained from the observation that Pool1 in solfatara Pisciarelli is surrounded by a rich and diverse vegetation that, dropping in the pool, might be a source of different (hemi)celluloses. CAZymes belonging to GH1 show several substrate specificities, including glycosides of glucose, galactose, xylose, mannose, glucuronic acid, fucose, phospho-glucose, and phospho-galactose all involved in β-O-glycosidic bonds frequently found in the plant (hemi)celluloses [42]. Enrichments on Arundo and Thistle biomasses, might have selected for GH1 CAZymes specific for the lignocellulose present in these sources.

A different trend was observed for families grouping enzymes active on axial C1-OR. A substantial increase in number was observed among sequences belonging to GH15, GH31, GH122, and GH133, and, remarkably, we could identify several sequences from GH109, a family not found in Pool1. Possibly, starch is more abundant in Arundo [23] than in the plant biomass present in Pisciarelli solfatara [27]. Alternatively, starch is more persistent at the conditions used for the enrichment if compared to those observed in vivo at very low pH and high temperatures. Therefore, specific CAZymes might have been selected for its hydrolysis and acted more efficiently than (hemi)cellulases on the lignocellulose component of Arundo and Thistle. However, it is worth noting that starch is a relevant component of these energy crops, which are often considered mainly as sources of lignocellulose.

**Figure 6:**
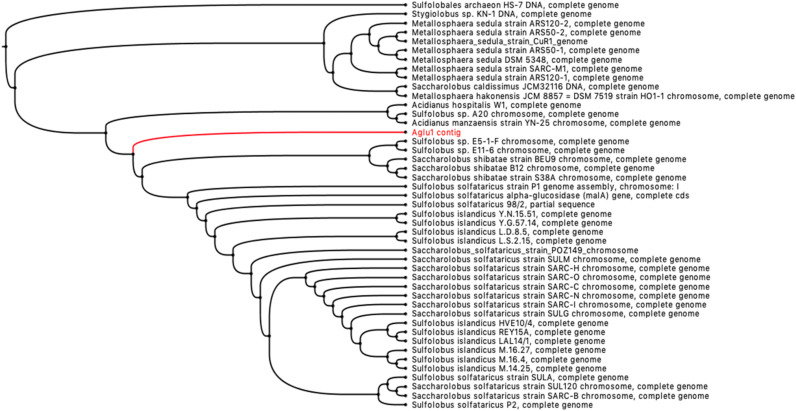
Phylogenetic blastx tree of the metagenomic contig containing the gene *aglu1* (branch in red).

The sequences annotated as putative GHs showed a wide range of identities towards the characterized GHs present in the CAZy database (www.cazy.org). In particular, although 19% have 100% identity with already characterized sequences, more than 50% of the sequences, identified in the enriched samples, represent completely new GHs showing identity ≤60% towards those characterized (Figure S10). Moreover, about 10% of the hypothetical GHs identified show an identity of 80% and 90%, respectively, compared to those characterized. This is still interesting, as is well-known that an amino acid sequence identity difference of even just 10% could entail substantial differences in terms of substrate specificity, stability, pH dependence, etc. [51,52,53,54]. Among this group of putative GHs, a sequence encoding a new uncharacterized archaeal enzyme, homolog to α-glucosidases belonging to GH31, has been identified. Although a high number of α-glucosidases belonging to the GH31 family have been isolated from Bacteria and Eukaryota, relatively few are known from Archaea. To date, only six α-glucosidases (EC 3.2.1.20) from thermophilic archaea have been characterized: AglA (PTO0092) from *Picrophilus torridus* DSM 9790 [37], MalA (Sso1_0793) from *Saccharolobus solfataricus* 98/2 [33], MalA (Saci_1160) from *Sulfolobus acidocaldarius* DSM639 [36], MalA (SSO3051) from *S. solfataricus* P2 [39], ST2525 from *S. tokodaii* str.7 [35], and AglA (Ta0298) from *Thermoplasma acidophilum* DSM 1728 [38]. The predicted product of the gene *aglu1* showed a sequence identity of 88% with a putative α-glucosidase of the hyperthermophilic crenarchaeon *Saccharolobus shibatae* (WP_240781539.1) and 82% with the characterized α-glucosidase from the hyperthermophilic crenarchaeon *S. solfataricus* MalA (SSO3051). It is worth noting that the analysis of the metagenomic contig containing *aglu1* gene, as well as four ORFs related to the maltose transport system MalK/MalG/MalF (Figure S4b), demonstrated its phylogenetic distance from the genera *Saccharolobus* and *Sulfolobus*. However, the relationship to the family of *Sulfolobaceae*, with a genomic environment similar to *S. solfataricus* P2 and *S. shibatae*, suggests that it belongs to a chromosome of a new, unclassified genus (Figure 6 and Figure S4).

The deduced protein sequence of Aglu1 was multi-aligned toward the archaeal characterized enzymes belonging to GH31, showing the presence of the conserved catalytic residues of this family, and suggesting that the gene encodes for a functional enzyme. Indeed, the recombinant pure Aglu1 was optimally active on 4Np-α-Glc in a sodium acetate buffer in the range of pH 3.5–5.0 (Figure 4a) at 95 °C (Figure 4c) showing remarkable stability at 75 °C, 85 °C and 95 °C (Figure 4d).

Aglu1 showed high activity on both α 1,4- and α 1,2-glycosidic bonds with a similar catalytic efficiency on both maltose and kojibiose (Table 1). It is worth noting that Aglu1, though being classified as a Type II α-glucosidase, since it hydrolyses preferentially maltose and small maltooligosaccharides over aryl-α-glycosides (Table 1) [55], is the first archaeal GH31 that is reported to be active on kojibiose, showing a catalytic efficiency on this substrate similar to that on maltose.

Compared to other characterized archaeal GH31, Aglu1 was the most active on 4Np-α-Glc (see the k_cat_ in raw 1 of Table 2) and preferentially hydrolyzed the α-1,4-glycosidic linkages within short chain length. It worth mentioning that k_cat_ of Aglu1 was 327 fold higher than that of ST2525 enzyme from *S. tokodaii*, although kinetic parameters of ST2525 were measured only at a temperature that was 5 °C lower than Aglu1 (80 °C vs. 85 °C) [35] (Table 2). Moreover, Aglu1 showed a k_cat_ ~20- fold higher than MalA from *S. acidocaldarius* on 4Np-α-Glc, although kinetic parameters of the latter enzyme were measured at 95 °C, only 5 °C below its optimal temperature [33]. Aglu1 was also the second most active enzyme on maltose and maltotriose after MalA from *S. acidocaldarius* (see the k_cat_ in raw 2 and 3 of Table 2) while it was completely inactive on 4NP-α-Xyl, differently from the GH31 XylS from *S. solfataricus* [34]. For its ability to catalyze the hydrolysis of maltodextrines at a high temperature, Aglu1 could be an interesting candidate to be included in the enzymatic cocktails used for the combined liquefaction/saccharification of starch, a well know application of α-glucosidases. In addition, its activity on kojibiose, a low caloric disaccharide, may offer the possibility of using Aglu1 synthesizing this sugar by transglycosylation or as a modified *glycosynthase* through an approach combining enzyme and reaction engineering [56,57,58].

Aglu1 and MalA from *S. solfataricus* shared a high percent of sequence identity (>80%) and the same oligomeric organization into a hexamer. In silico molecular modelling on MalA structure revealed that Aglu1 overall structure is also conserved, except for the domains C and D which were the regions that displayed the lowest identity (50% MalA) (Table S3). As reported in [39], these domains were probably gained early in the evolution of GH31 and subsequently, they diverged considerably by conferring specialized properties and being the main driving force for the different enzymatic activities found in the GH31 family [39]. A detailed structural characterization of Aglu1 goes beyond the aims of this work, but, possibly, the differences observed in domains C and D between Aglu1 and MalA may be responsible for the k_cat_ that is 10- and 2-fold higher in Aglu1 vs. MalA, on 4Np-α-Glc and maltose, respectively.

In this study, we demonstrated that samples taken from solfataric environments can be used to select a microbial consortium able to grow efficiently in a lab on biomasses from energy crops. Instead of a classical selection of new strains, our metagenomic approach was innovative and efficient. The analysis of the metagenomic data on three enrichments allowed for a global view of the microbial composition of the selected consortia and to identify many novel genes encoding for potential GHs. Surprisingly, the selection produced a number of genes encoding for CAZymes potentially active on starch that was higher than those potentially active on (hemi)cellulose. In addition, the expression and characterization of one of these genes allowed to identify a novel α-glycosidase, from a novel unknown archaeon, that was the first GH31 able to convert efficiently kojibiose into glucose.

## 4. Materials and Methods

### 4.1. Materials

All commercially available substrates were purchased from Merck, Carbosynth and Megazyme. The synthetic oligonucleotides were from Eurofins (Italy). The pretreated biomass of *A. donax* used in this study was derived from a pretreatment step by PROESA^®^ technology of the Chemtex Group. *C. cardunculus* used in this study was provided by Novamont. Both biomasses were provided in the frame of the project “PON 01_0966 ENERBIOCHEM”.

### 4.2. Environmental Sampling

The sample from the hydrothermal mud/water Pool1 in Pisciarelli solfatara was collected into sterile bottles and immediately transferred to the laboratory for in vitro cultivation. In situ measurements of temperature and pH were performed by using a HI-93510 thermometer (HANNA instruments, Padova, Italy) equipped with a Pt100 probe and litmus tests. Next, pH was accurately measured again with a pH meter (Crison Instruments, Inc., Barcelona, Spain) in the laboratory.

### 4.3. Microbial Enrichment and Isolation of Metagenomic DNA

For the enrichment setup, three aliquots of the environmental sample were grown in three different basal nutrient media, selected based on microbial consortium populating Pisciarelli solfatara: Basal Salt Medium (BSM) at pH 1.8 [29], Brock salt medium pH 3.5 [30], and Pyrobaculum (PYR) salt medium pH 7.0 [31], supplemented by tryptone, sucrose and yeast extract 0.1% as initial carbon source. Every three days, serial dilution (1:5 *v*/*v*) of the cultures in fresh salt medium with pretreated *Arundo donax* (0.15% *w*/*v*) was performed. Contextually, a control culture was performed by using trypton, sucrose and yeast extract 0.1% as carbon source.

For the enrichment experiment, the environmental sample was grown on Brock salt medium pH 3.5 [30] supplemented by tryptone, sucrose and yeast extract 0.1% as initial carbon source. After three days, the culture was split into three aliquots and diluted 1:5 *v*/*v* in a fresh salt medium with three different biomasses each one: pretreated *A. donax*, woodchips *A.donax*, woodchips *Cynara cardunculus*. Every three days, serial dilution 1:5 *v*/*v*) of the cultures in fresh salt medium with biomasses was performed. Contextually, a control culture was set up by using tryptone, sucrose and yeast extract 0.1% as carbon source.

The enriched microbial populations were recovered by centrifugation at 5000 g for 20 min at RT. The DNA from the enriched microbial population was extracted with Power Soil DNA Isolation Kit (MO BIO Laboratories, Inc., Carlsbad, CA, USA) by following the manufacturer’s protocol.

The extracted and purified DNA from enriched samples was used for shotgun sequencing with HISeq2000 (Illumina) performed at Beijing Genomics Institute (BGI-Shenzhen) Shenzhen, China.

The sequencing reads are available in the NCBI Sequence Read Archive (SRA) database in the Bioproject PRJNA861126 under the accession numbers SAMN29886219 (YTS), SAMN29933369 (Arundo woodchip), SAMN29933377 (Arundo pretreated), and SAMN29933397 (Thistle woodchips).

### 4.4. Taxonomic Analysis and Functional Annotation

For microbial diversity analysis, short paired-end Illumina reads (90 bp) of each sample were analyzed by using Kaiju against the NCBI RefSeq protein database [59].

Clean reads were assembled using MEGAHIT [60] in “meta-sensitive” mode by using min-count = 2 and k-mers 21, 31, 41, 51, 61, 71, 81, 91, and 99.

All the contigs obtained by the assembly were analyzed by using Prodigal [61] to identify the open reading frames and analyzed by dbCAN2 pipeline for the carbohydrate-active enzymes annotation [32].

The ORFs assigned as glycosidases were analyzed by using Diamond in bastp mode [62], against a custom protein database with the 7190 sequences of the characterized GHs from the CAZy database [28].

The contig encoding Aglu1 and the Aglu1 sequence are available in the GenBank database under the accession numbers OP149530 and OP149529, respectively.

### 4.5. Cloning, Expression, and Purification of Recombinant Aglu1

The aglu1 gene was amplified by PCR from the metagenome DNA extracted from enrichment on pretreated *A. donax* using the synthetic oligonucleotides GH31_forward (5′-GGAATTCCATATGCAAGTAACAAAGATATACGAGAG-3′) and GH31_reverse (5′-CCGCTCGAGCCTTCTTAAGTTAATATTTTTATCC-3′).

The amplification reaction was performed with the PfuUltra Ultra HF DNA Polymerase (Stratagene) by using the following program: hot start 5 min at 95 °C; 5 cycles 1 min at 95 °C, 1 min at 50 °C and 1.5 min at 72 °C; 30 cycles 1 min at 95 °C, 1 min at 60 °C, and 1.5 min at 72 °C; final extension 10 min at 72 °C. The DNA fragment obtained was cloned in the expression vector pET20b (Invitrogen, Waltham, MA, USA), obtaining the recombinant plasmid pET20b-Aglu1. The PCR-generated construct was verified by sequencing and the ORF was expressed in Escherichia coli cells, strain BL21 (DE3) RIL (Invitrogen), according to the manufacturer. The cells transformed with pET20b-Aglu1 were grown at 37 °C in 2 L of Super Broth at 37 °C supplemented with ampicillin (50 µg mL^−1^) and chloramphenicol (30 µg mL^−1^). Gene expression was induced by the addition of 0.5 mM IPTG when the culture reached an A600 of 0.6 OD. Growth was allowed to proceed for 16 h, and cells were harvested by centrifugation at 5000× *g*. The resulting cell pellet was resuspended in 50 mM sodium phosphate buffer, pH 8.0, 300 mM NaCl and 1% TRITON-X100 with a ratio of 3:1 (*v*/*w*) and then incubated at 37 °C for 1 h with 20 mg of lysozyme (Fluka) and 25 U g^−1^ cell of Benzonase (Novagen, Madison, WI, USA). Cells were lysed by French cell pressure treatment and cell debris was removed by centrifugation at 12,000× *g* for 30 min. The free cellular extract (FCE) was heat-fractionated three times (i) for 30 min at 50 °C, (ii) 30 min at 60 °C, and (iii) for 30 min at 70 °C. The resulting supernatant was loaded on Q Sepharose High Performance 16/10 (GE-Healthcare, Chicago, IL, USA) equilibrated with Tris HCl 20 mM pH 8.0 (Buffer A). After an initial wash-step (2-column volumes, CV) with buffer A, the protein was eluted with a 3-CV gradient up to 100% of Tris HCl 20 mM pH 8.0 + 1 M NaCl (Buffer B), followed by 2-CV of Buffer B at a flow rate of 2 mL min^−1^. The active fractions were pooled and dialyzed against Tris HCl 20 mM pH 8.0. (NH_4_)_2_SO_4_ 1 M was added to dialysed pool successively loaded on Phenyl Sepharose High Performance 26/10 (GE-Healthcare) equilibrated with Tris HCl 20 mM pH 8.0 + (NH_4_)_2_SO_4_ 1 M (Buffer A). After an initial wash-step (1.5 CV) with buffer A, the protein was eluted with a 5 CV gradient up to 100% of Tris HCl 20 mM pH 8.0 (Buffer B), followed by 2 CV of Buffer B at a flow rate of 3 mL min^−1^. The active fractions were pooled and dialyzed against Tris-HCl 20 mM pH 8.0 and concentrated by ultrafiltration on an Amicon YM30 membrane (cut off 30,000 Da).

### 4.6. Molecular Mass Determination

The molecular mass of Aglu1 was determined by gel filtration on a Superose 6 10/300 FPLC column (GE-Healthcare) with an isocratic solution of 20 mM Tris HCl (pH 8.0). Molecular weight markers were Thyroglobulin (669 kDa), apoferritin (443 kDa), Bovin Albumin (BSA) (66 kDa) and Cytochrome C (12.7 kDa).

### 4.7. Standard Assay

The standard assay for Aglu1 was performed on 5 mM 4Np-α-Glc in 50 mM sodium acetate buffer at pH 4.5 at 65 °C by using 2 µg of enzyme in the final volume of 0.2 mL. After 1 min of incubation at 65 °C, the reaction was blocked in ice and by adding 0.8 mL of 1 M sodium carbonate pH 10.2. The absorbance was measured at 420 nm at room temperature; the mM extinction coefficient of 4-nitrophenol in this condition is 17.2 mM^−1^ cm^−1^. In all the assays, spontaneous hydrolysis of the substrate was subtracted by using appropriate blank mixtures without the enzyme. One unit of activity was defined as the amount of enzyme catalyzing the conversion of 1 μmole of substrate into the product in 1 min, at the conditions described.

### 4.8. Temperature and pH Influence

The temperature and pH optima were determined by assaying Aglu1 in 50 mM of the indicated buffers at different pHs in the range of 40–100 °C in the standard assay conditions. Thermal stability was evaluated by incubating the enzymes in Tris HCl 20 mM pH 8.0, at the indicated temperatures (75 °C, 85 °C, 95 °C). At intervals, aliquots (2 µg of enzyme) were withdrawn, transferred in ice, centrifuged for 1 min at 16,000× *g* and assayed at standard conditions. The residual activities were expressed as a percentage of the maximal enzymatic activity measured before the incubation at indicated temperatures.

All the experiments were performed in duplicate.

### 4.9. Substrate Specificity and Steady-State Kinetic Constants

The activity of Aglu1 was tested on different aryl-glycosides, oligosaccharides (maltose, maltotriose, maltotetraose, maltopentaose, trehalose, and nigerose), and polysaccharides (starch, and glycogen) in 50 mM sodium acetate buffer at pH 4.5 and 65 °C for 1 h. D-Glucose released by the hydrolysis of oligo- and polysaccharides can be measured by using Megazyme D-Glucose GOPOD kit according to manufacturing protocol.

To define the mode of action (*endo-* versus *exo-*acting), Aglu1 was tested on maltodextrins ranging from two to five glucose residues. The products were analyzed on silica gel 60 F254 TLC by using acetone/isopropanol/water (60:30:15 v:v) as eluent and 5% sulfuric acid in methanol for detection.

Kinetic constants of Aglu1 on 4Np-α-Glc and maltose were measured in 50 mM sodium acetate buffer at pH 4.5 at 85 °C by using substrates ranging from 0.5 to 35 mM and from 0.2 to 30 mM, respectively. Spontaneous hydrolysis of the substrates was subtracted by using appropriate blank mixtures without enzyme.

Kinetic constants of MalA on maltose were measured in 100 mM sodium citrate buffer at pH 5.0 at 85 °C. Spontaneous hydrolysis of the substrates was subtracted by using appropriate blank mixtures without enzyme. All kinetic data were calculated as the average of at least two experiments and were plotted and refined with the program Prism 5.0 (GraphPad Software, San Diego, CA, USA).

## 5. Conclusions

The metagenomic analysis of cultures of hyperthermophilic microorganisms enriched on plant biomasses allowed the identification of several genes encoding for GHs still unclassified and uncharacterized, and potentially useful for biotechnological application. The use of vegetable wastes as culture media for consortia of extremophilic microbes can represent a cheap carbon source to achieve two promising targets: a method of waste handling at lower environmental impact and the possibility to select extremozymes of biotechnological interest using cheap feedstocks not competing with food supplies.

Indeed, enrichments on two specific energy crops showed that a remarkable selection toward a high number of diverse carbohydrate active enzymes. Surprisingly, the selection was more efficient on starch-degrading enzymes rather than on biocatalysts specific on lignocellulose. The enrichments allowed us to identify and characterize a novel, highly thermophilic and thermostable archaeal α-glucosidase, whose genetic organization suggests that it belongs to a new member of the *Sulfolobaceae*. Aglu1 hydrolyses maltose and small maltooligosaccharides with specificity constants higher than those observed on aryl-glucosides, and an unexpected specificity on kojibiose. The enzymatic characterization demonstrated that Aglu1 is the archaeal GH31 most active on 4Np-α-Glc and one of the most active enzymes from this source on maltose.

This study demonstrated that in-lab enrichments of microbial communities from extreme environments represent an efficient strategy for the identification of novel thermophilic and thermostable CAZymes and that energy crops work as rich media allowing the identification of enzymes able to act cooperatively to produce fermentable sugars not only from lignocellulose, but also from starch.

## Figures and Tables

**Table 1:** Steady-state kinetic constants of Aglu1.

Substrates	V_MAX_ (U mg^−1^)	K_M_ (mM)	k_cat_ (s^−1^)	k_cat_/K_M_ (s^−1^ mM^−1^)
4Np-α-Glc	42.5 ± 1.1	3.59 ± 0.33	327.4 ± 8.4	91.14
Maltose	117.1 ± 6.9	3.16 ± 0.65	901.6 ± 53.6	284.97
Maltotriose	89.7 ± 3.2	1.03 ± 0.20	690.7 ± 24.4	670.64
Kojibiose	65.0 ± 1.9	1.41 ± 0.19	500.2 ± 14.6	352.49

**Table 2:** Comparison of the steady-state kinetic constants of Aglu1 with all archaeal characterized GH31. ^a^ (This study), ^b^ [56], ^c^ [37], ^d^ [36], ^e^ [35], ^f^ [38]. Kinetic Parameters of Aglu1, MalA from *S. solfataricus* and AglA from *P. torridus* were measured at 85 °C, while MalA from *S. acidocaldarius*, ST2525 from *S. tokodaii* and AglA from *T. acidophilum* at 95 °C, 80 °C and 80 °C, respectively. ND: not detected.

	Substrate	Kinetic Parameter	Aglu1 ^a^	MalA ^b^ *S. solfataricus*	AglA ^c^ *P. torridus*	MalA ^d^ *S. acidocaldarius*	ST2525 ^e^ *S. tokodaii*	AglA ^f^ *T. acidophilum*
1	4Np-α-Glc	K_M_ (mM)	3.59 ± 0.33	1.7 ± 0.2	0.94 ± 0.14	0.87 ± 0.18	3.18	1.77
		k_cat_ (s^−1^)	327.4 ± 8.3	38.5 ± 1.6	65.1	16.7	1.05	59.7
		k_cat_/K_M_ (s^−1^ mM^−1^)	91.14	22.1	69.25	19.2	0.33	33.7
2	Maltose	K_M_ (mM)	3.16 ± 0.65	2.00 ± 0.12 ^a^	3.70 ± 0.32	0.80 ± 0.32	10.9	0.65
		k_cat_ (s^−1^)	901.6 ± 53.5	481.1 ± 6 10^−5 a^	188.1	1277.8	6.5	143
		k_cat_/K_M_ (s^−1^ mM^−1^)	285.0	239.6 ^a^	50.83	1597.2	0.598	220
3	Maltotriose	K_M_ (mM)	1.03 ± 0.20	ND	ND	0.30 ± 0.08	3.11	0.47
		k_cat_ (s^−1^)	690.7 ± 24.4			550	2.53	90.2
		k_cat_/K_M_ (s^−1^ mM^−1^)	670.64			1833.3	0.814	194

## Data Availability

Not applicable.

## Supplementary Materials

The following supporting information can be downloaded at: https://www.mdpi.com/article/10.3390/ijms231810505/s1.
