# Supplementary material for: Valorization of Biomasses from Energy Crops for the Discovery of Novel Thermophilic Glycoside Hydrolases through Metagenomic Analysis
Source: Int J Mol Sci. 2022 Sep 10;23(18):10505. doi: 10.3390/ijms231810505 (PMC9505709; doi:10.3390/ijms231810505)
Supplement: Supplementary file 1 [file ijms-23-10505-s001.zip › Iacono et al_IJMS-1883995_Supplementary figures.pdf]

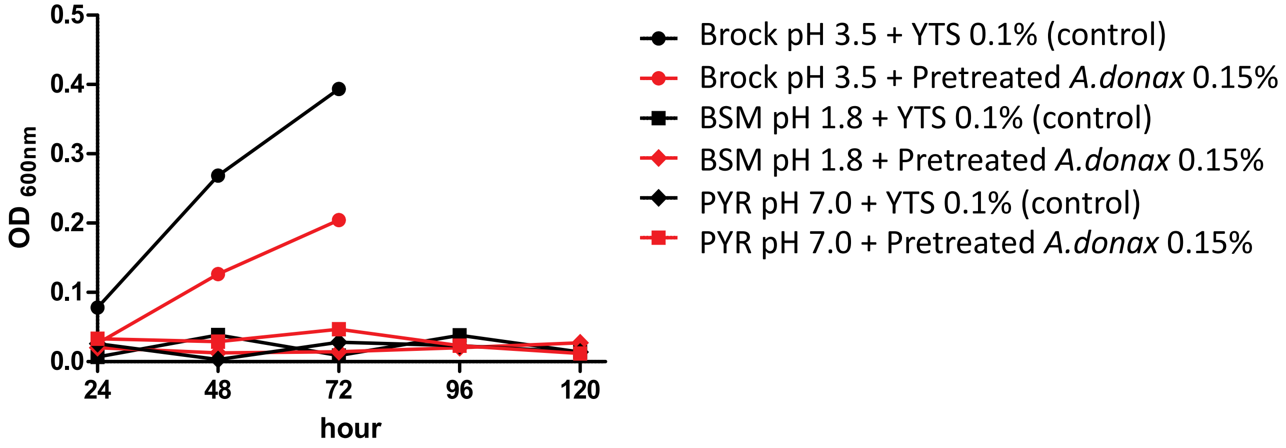

Figure S1. Microbial growth curves of the enrichments on pretreated *A. donax* in different culture media.

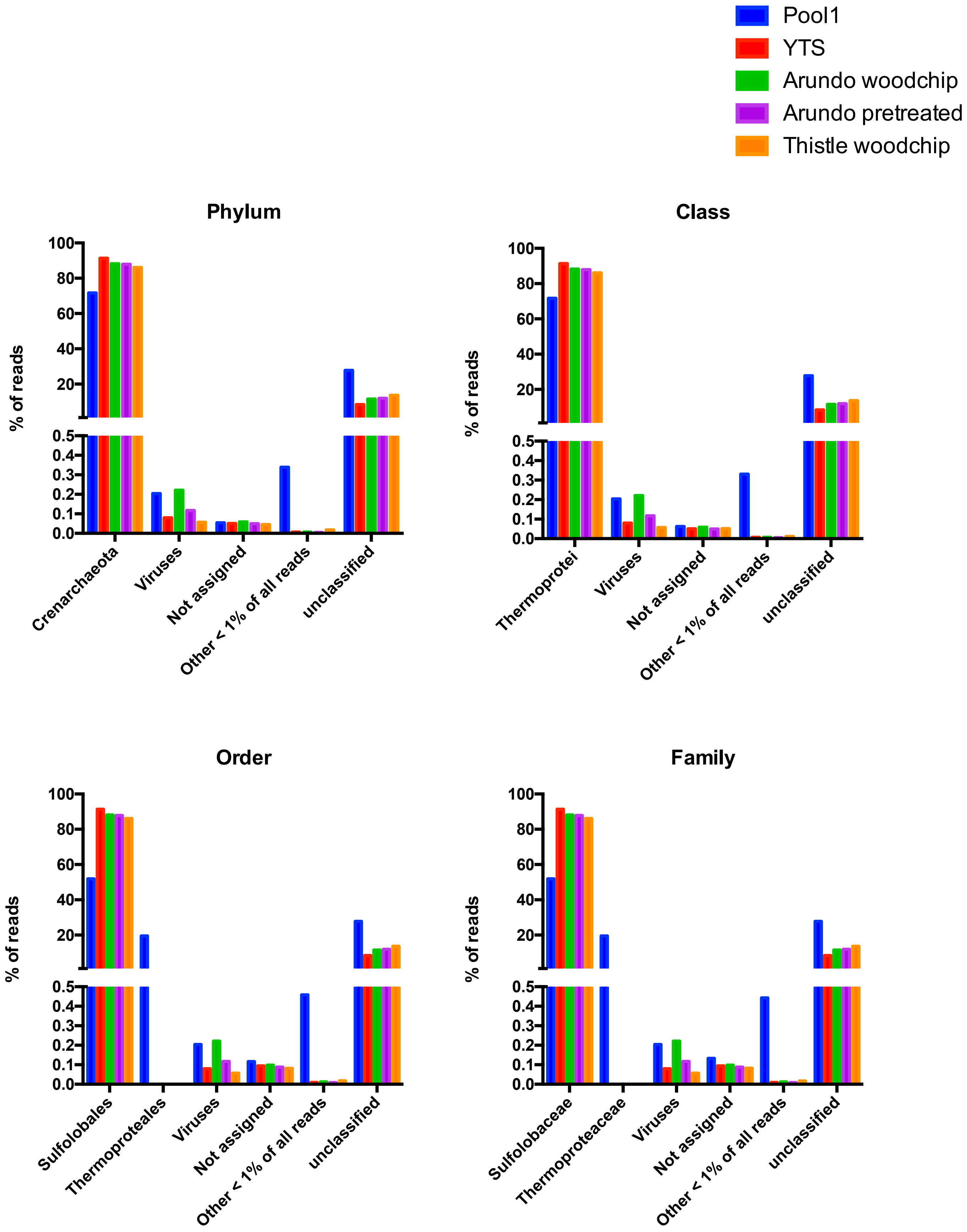

Figure S2. Taxonomic annotation at the level of phylum, class, order, and family.

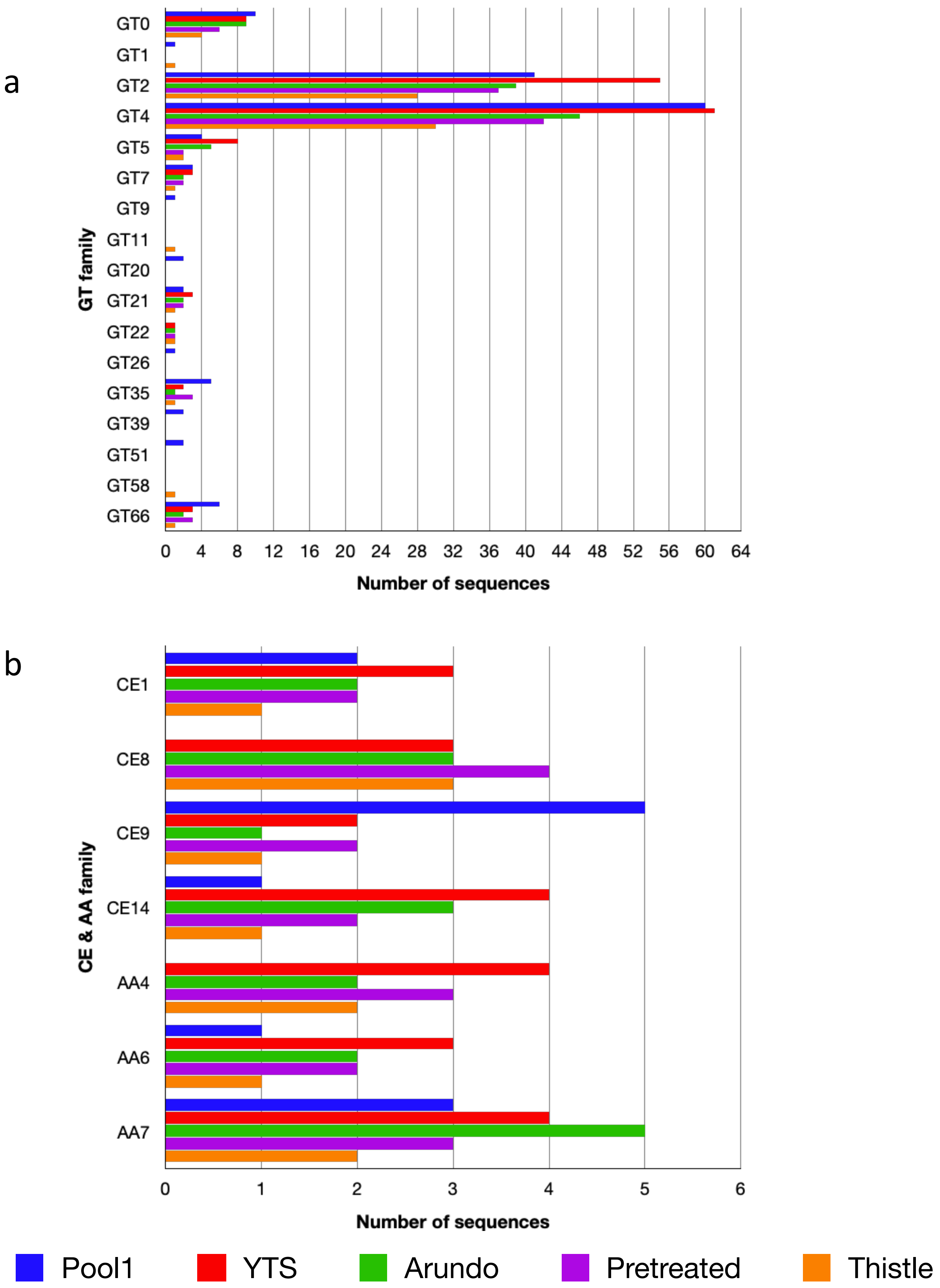

Figure S3. Distribution among the samples of the (a) glycosyltransferase, (b) carbohydrate esterases and auxiliary activities. The number of sequences assigned to each family from Pool1 and the four enrchments is displayed.

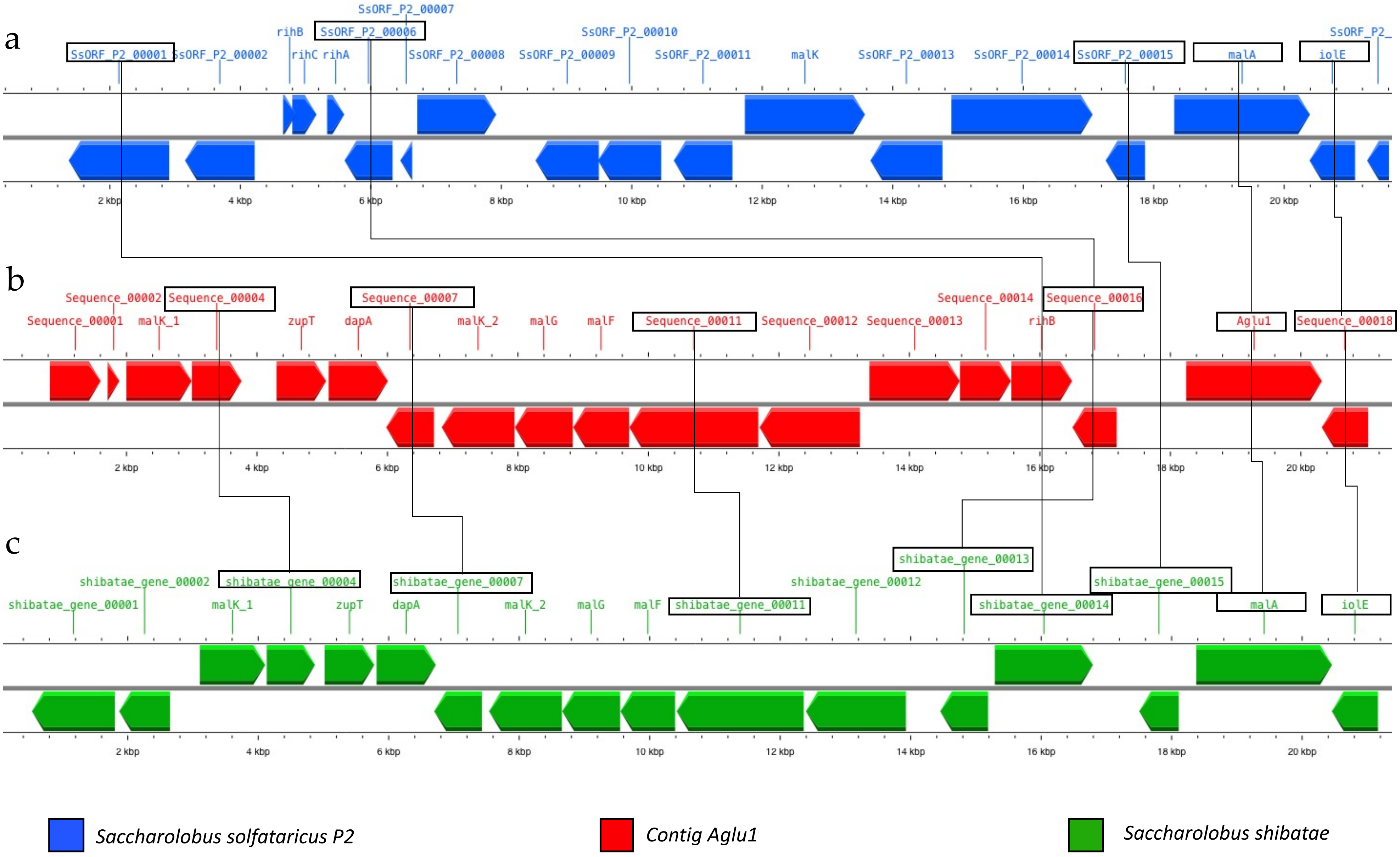

Figure S4. Genomic environments of *S. solfataricus P2 malA* (a), the contig containing *aglu1* gene (b) and and *S. shibatae* malA (c). Calluts and connectors indicate homologous unannotated ORFs with amino acid identity ≥ 80%

```
SSo3051         --KFCEIESGELFVGKMWPGTTVYPDFFREDTREWWAGLISEWLSQG-VDGIWLDMNEPT 325
Aglu1           --KFCEIETGELFVGKMWPGTTVYPDFFREDTREWWAGLISEWLSQG-VDGIWLDMNEPT 325
ST2525          --KYCETDKGELFVGKLWPGNSVYPDFFREETRKWWSELISKWLSQG-IDGIWLDMNEPT 318
Saci_1160       --NYVENEDGTIYADILWPGLSVFPDFLNSKTREWWRNLVEKWVKENNIDGIWLDMNEPS 323
PTO0092         --KYVINSNNEIYISRLWPGNCAFLNFLDADSYNYWKSCVKEFAENV--DGIWLDMNEPA 319
Ta0298          GGYFVKYPDGNVMYAPVWPEMAAFPDFTDEKAREWYASKYDFMRSMG-VSGFWHDMNEPA 413
XyLS            ---------------CIILGTTAFNPFKDECRELFWS-YVKGFYDLG-IDAYWLDASEPE 358
                                :     .:  *       ::    .   .    .. * * .**

SSo3051|        KGFRTSHRNEIFILSRAGYAGIQRYAFI-WTGDNTPSWDDLKLQLQLVLGLSISGVPFVG 443
Aglu1           EGFRKSGRSEVFILSRAGYAGIQRYAFI-WTGDNTPSWDDLRLQLQLVLGLSISGIPFVG 443
ST2525          EGFK--NKEEVFILTRGGYAGIQKFAGI-WTGDNTPSWDDLKLQLQLILGLSISGIAYIG 422
Saci_1160       PSVD-------FVLSRAGYSGIQRYAAI-WTGDNTTSWSDLTLQLALTLGLSISGVPYVG 414
PTO0092         EALK-EIKDEFFILSRSGYPGIQRYAAI-WTGDNKASDDDLKLQISMIVSMNLSGIMICG 416
Ta0298          DHLS--KVERPFILSRSGWAGISRYGWI-WTGDTETSWKELKQNIITIMHMSMSGITLTG 507
XyLS            EGQRRISNKRVVILTRSAFAGQQRHSAISWSGDVLGDWATLRAQIPAGLNFSISGIPYWT 455
                           .:*:*..:.* .:.. * *:**   .   *  ::   : :.:**:
```

Figure S5. Multialignment of the regions close to catalytic residues (in red) of the amminoacidic sequences of the GH31 enzymes: SSo3051 MalA from *S. solfataricus,* Aglu1 (in red), ST2525 from *S. tokodaii* str.7, Saci_1160 MalA from *S. acidocaldarius DSM639,* PTO0092 AglA from *P. torridus DSM 9790,* Ta0298 AglA from *Thermoplasma acidophilum DSM 1728 and* XylS (Sso3022) from *S. solfataricus P2.*

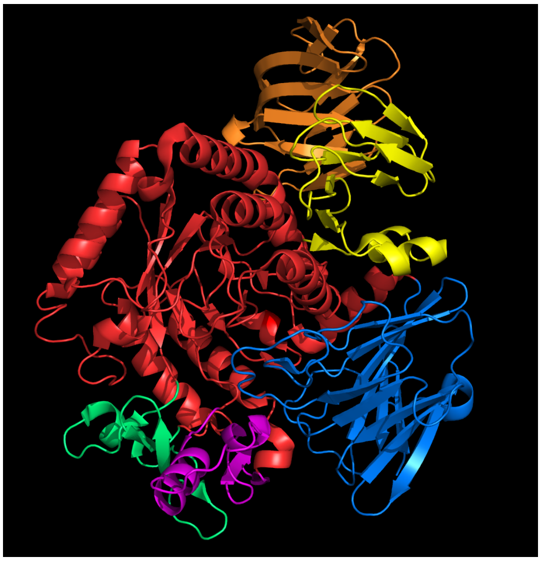

(a)

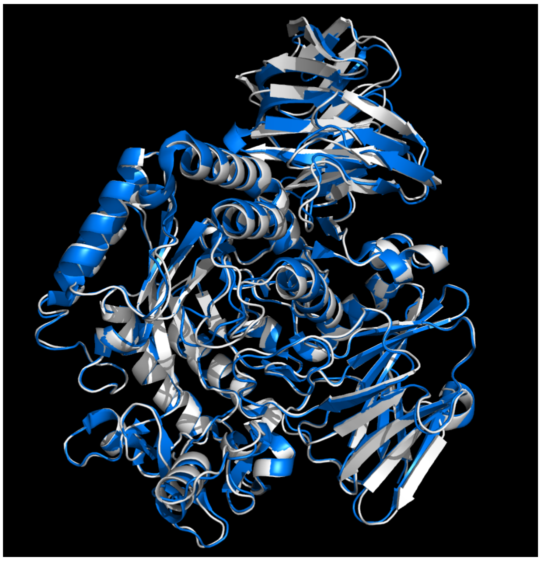

(b)

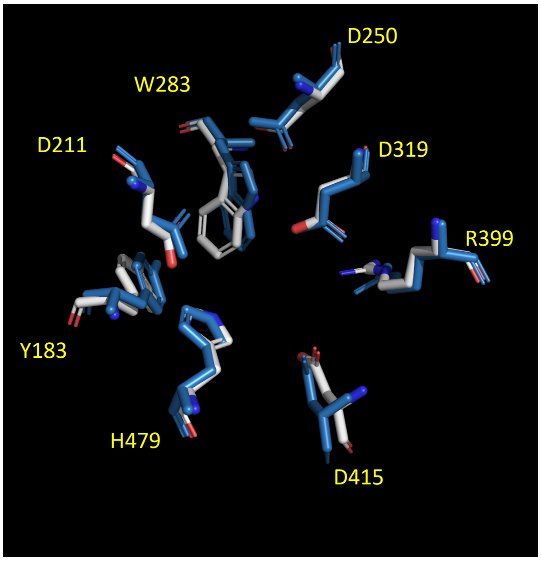

(c)

Figure S6 . a) Different domains and subdomains of *S. solfataricus* MalA 3D structure (PDB ID 2G3M). The different colours indicate the domains N (blue), A (red), C (yellow), D (orange), and subdomains B (green) and B′ (purple). b) Overall superimposition of S. solfataricus MalA (blue) and Aglu1 model (white). c) Close-up of the superimposed aminoacid residues in the active sites of the Aglu1 model (white) and MalA (blue).

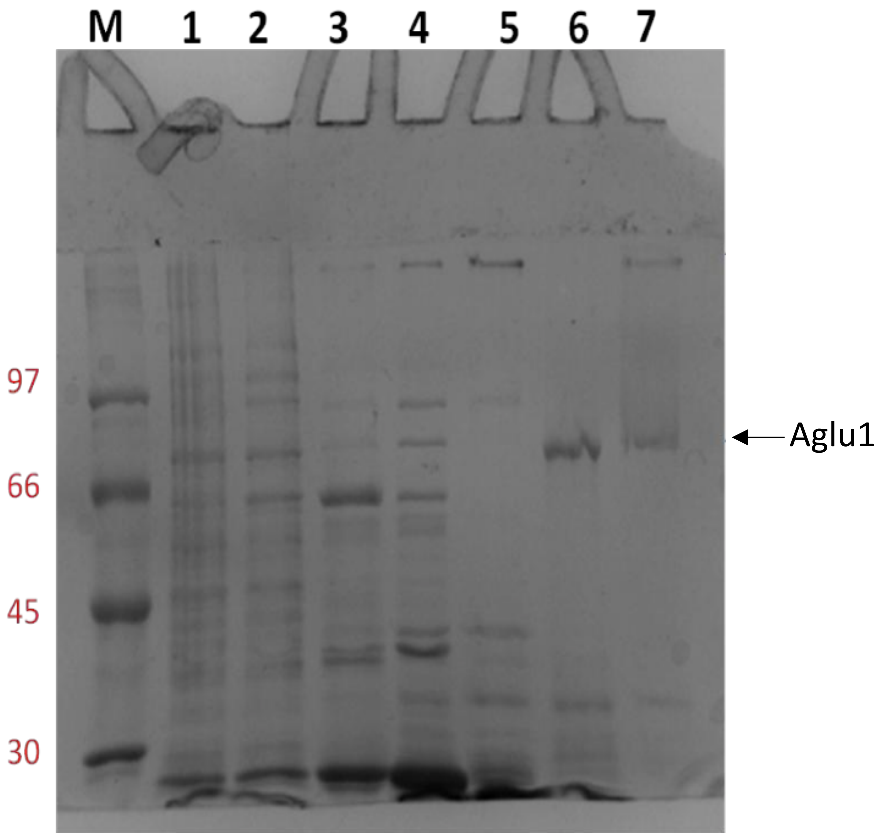

Figure S7 Purification steps of Aglu1. Lane M, molecular weight markers, lane 1) crude extract (7 µg); lanes 2–5) heat treatment at 50 °C (7 µg), 60 °C (10 µg), 70 °C (10 µg) and 80 °C (10 µg) respectively; lane 6, Q-Sepharose 16/10 (5 µg); lane 7, Phenyl Sepharose 26/10 (3 µg).

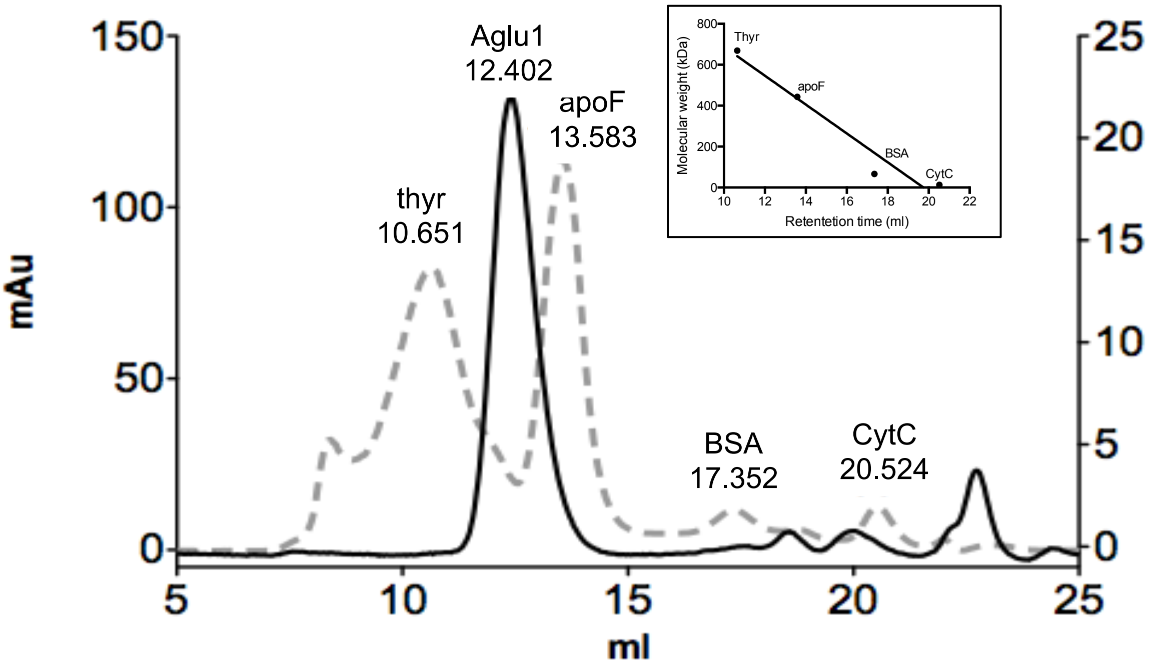

Figure S8. Elution profile of Aglu1 and molecular weight markers. Inset: graph of the calibration line obtained by plotting the retention time and the molecular weight.

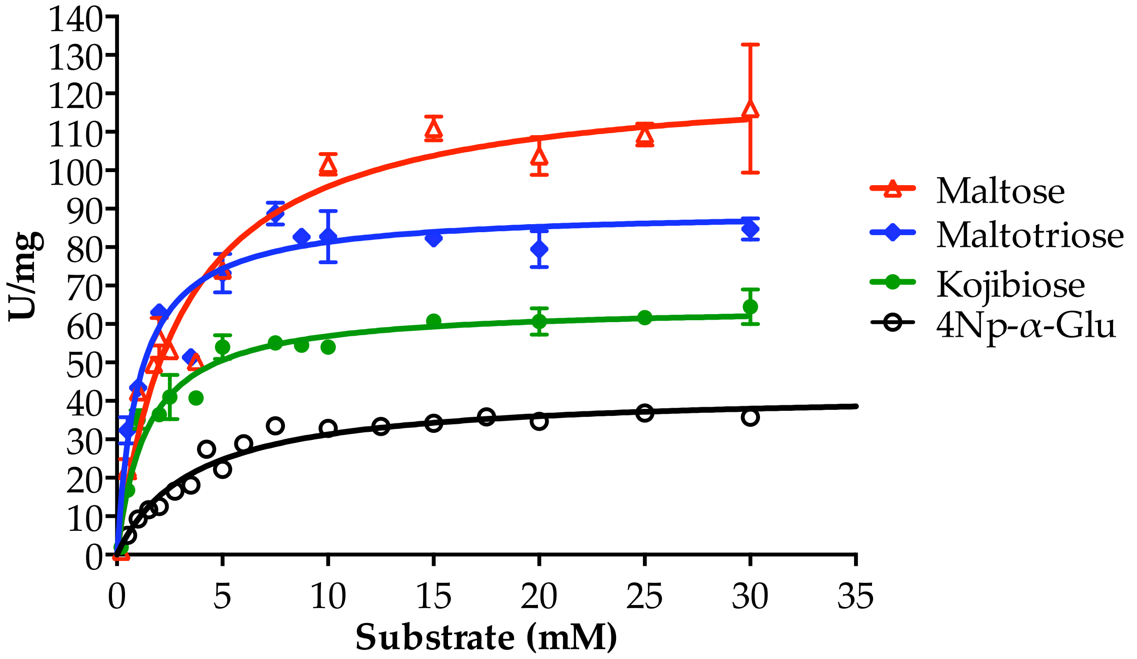

Figure S9. Kinetic curves of Aglu1 on, Maltose, Maltotriose, Kojibiose and 4Np-α-Glu.

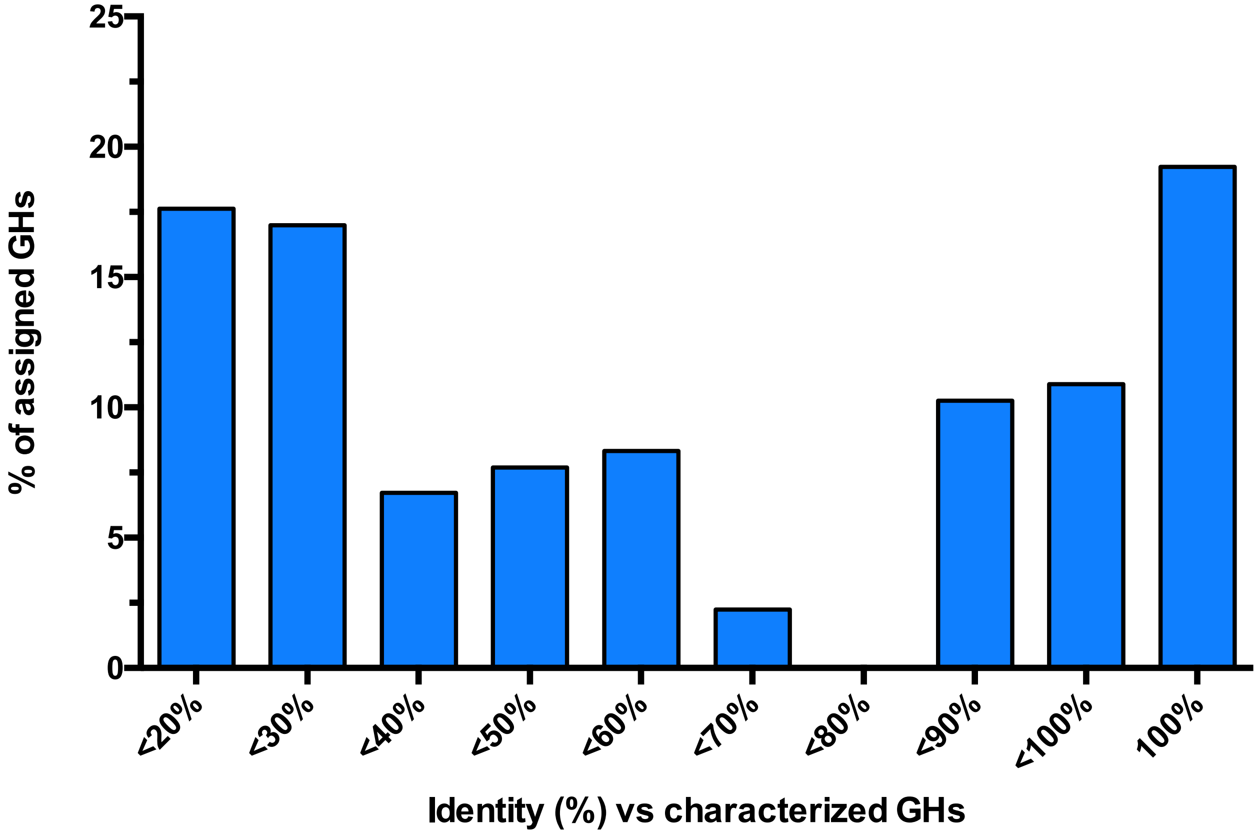

Figure S10. Percentage of the assigned GHs in the samples against the GHs characterized from www.cazy.org
