# Supplementary material for: Valorization of Biomasses from Energy Crops for the Discovery of Novel Thermophilic Glycoside Hydrolases through Metagenomic Analysis
Source: Int J Mol Sci. 2022 Sep 10;23(18):10505. doi: 10.3390/ijms231810505 (PMC9505709; doi:10.3390/ijms231810505)
Supplement: Supplementary file 1 [file ijms-23-10505-s001.zip › Iacono et al_IJMS_supplementary tables.pdf]

Table S1: Sequencing and assembly statistics

	Rich	Arundo	Pretreated	Thistle
Reads	22,222,224	22,222,224	22,222,224	24,444,446
Contigs ≥ 500 bp	1,338	1,751	1,368	549
Contigs ≥ 1,000 bp	948	1,110	901	426
Contigs ≥ 5,000 bp	353	213	177	158
Contigs ≥ 10,000 bp	161	81	86	87
Contigs ≥ 25,000 bp	48	27	26	29
Contigs ≥ 50,000 bp	20	12	10	10
Largest contig	489,871	185,195	179,766	167,948
Total length	8,138,047	5,734,578	4,976,681	3,768,893
N50	16,553	6,742	10,427	20,619
N90	2,397	1,164	1,212	2,844
auN	69,787	26,771	31,605	39,942
L50	80	138	84	42
L90	605	998	760	259
Total ORFs	10,161	8,416	6,535	4,383
Unique ORFs	9,947	8,334	6,439	4,300

Table S2: Purification of Aglu1

Purification steps	Total protein (mg)	Total activity (U)	Specific activity (U/mg)	Yield (%)	Purification fold
Cell extract	844				
50°C	708	70	0.10	100	1
60°C	180	63	0.35	90	3.5
70°C	48	57	1.2	81	12
80°C	21	52	2.5	75	25
Q- Sepharose	19	54	3	77	30
Phenyl- Sepharose	2.5	24	9.5	34	95

Table S3: Percent of sequence identity of each domain, comparing Aglu1 with MalA (SSO3051) from *S. solfataricus* and SSaci1160 from *S. acidocaldarius*.

	Aglu1 vs MalA (% identity)	Aglu1 vs Saci1160 (% identity)
Domain A	80	54
Domain N	88	48
Domain B	90	36
Domain B′	86	30
Domain C	68	49
Domain D	50	21
